# Arthroscopic Resection of Symptomatic Ossicles of the Medial Malleolus Combined With Deltoid Ligament Repairs in Athletes: A Report of Three Cases

**DOI:** 10.7759/cureus.64521

**Published:** 2024-07-14

**Authors:** Hiroki Yabiku, Hideaki Nagamoto, Yasunori Tome, Kotaro Nishida, Tsukasa Kumai

**Affiliations:** 1 Department of Orthopedic Surgery, Graduate School of Medicine, University of the Ryukyus, Okinawa, JPN; 2 Department of Orthopedic Surgery, Tohoku University, Sendai, JPN; 3 Department of Sports Medicine, Graduate School of Sport Sciences, Waseda University, Saitama, JPN; 4 Department of Orthopedics, Faculty of Sport Sciences, Waseda University, Saitama, JPN

**Keywords:** arthroscopy, case report, deltoid ligament, ligament repair, medial malleolus, athletes, os subtibiale, secondary ossification center, ankle

## Abstract

We report three cases of young athletes with symptomatic ossicles of the medial malleolus who underwent arthroscopic resections of the ossicle combined with deltoid ligament repair. A differential diagnosis of the ossicles beneath the medial malleolus, accessory ossification center, avulsion fracture resulting in pseudoarthrosis, and accessory bone such as os subtibiale has been proposed. However, it is difficult to differentiate them clearly. Most of these ossicles are asymptomatic, although they can cause chronic medial ankle pain, especially in young athletes who require surgical treatment. All three patients had pain in the distal part of the medial malleolus, which restricted their sports activities. Plain radiographs of all three cases revealed a well-defined, round-shaped bony lesion beneath the medial malleolus. Ultrasonographic imaging, magnetic resonance imaging, and arthroscopic findings revealed that ligament attachment to the ossicle varies in volume and type according to the cases. In other words, the mechanisms through which the existence of the ossicles affects the stability of the ankle joint and foot alignment are different in each case, indicating that deltoid ligament repair is necessary according to the cases. In all three cases, we performed arthroscopic resections of ossicles combined with deltoid ligament repairs, achieving favorable short-term clinical outcomes.

## Introduction

Ossicles beneath the medial malleolus are occasionally observed. Although most ossicles are asymptomatic, they can cause chronic medial ankle pain, particularly in young athletes [1-4]. Accessory ossification centers, avulsion fractures resulting in pseudoarthrosis, and accessory bones such as the os subtibiale have been proposed as differential diagnoses of ossicles beneath the medial malleolus. Although differentiation of the origin of ossicles by morphology and location has been reported [5], it remains challenging to distinguish between them. However, regardless of which disease caused the ossicles, patients who do not respond to conservative treatment may require surgery. Several surgical treatments for symptomatic ossicles of the medial malleolus have been reported [2,3,6-8]. However, the optimal surgical treatment remains unclear.

Here, we report three cases of symptomatic ossicles of the medial malleolus treated with arthroscopic resection of the ossicles combined with deltoid ligament repair.

## Case presentation

All three cases in the present report underwent surgery at Hanna Chuo Hospital, where authors HY, HN, and TK are currently affiliated or were previously affiliated. In the present cases diagnosed with symptomatic ossicles of the medial malleolus, ultrasonographic imaging, magnetic resonance imaging (MRI), and arthroscopy revealed that the ossicles involved a relatively wide attachment site of the deltoid ligament. Therefore, the arthroscopic resection of the ossicles combined with deltoid ligament repair was performed to achieve favorable clinical outcomes.

**Figure 1:**
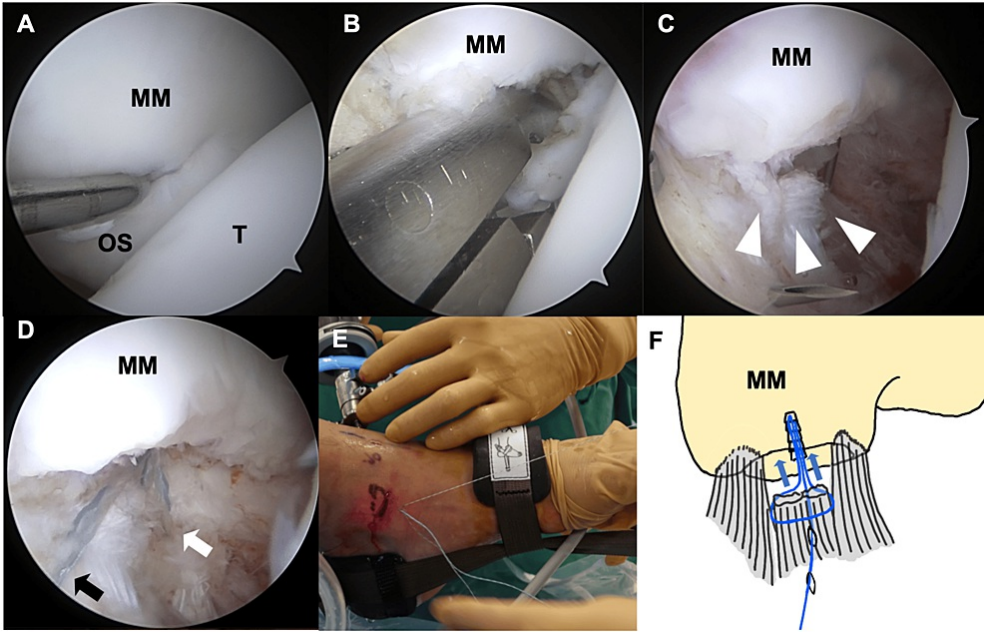
Surgical procedures (A) Border between the medial malleolus (MM) and the ossicle (OS) can be identified by depression and softening of cartilage. (B) Resection of the OS. (C) The proximal end of detached deltoid ligament (arrowheads). (D, E) Suture wire is passed through the anterior edge of the detached ligament (black arrow), subcutaneous tissue along with the outer surface of the capsule, and posterior edge of the detached ligament (white arrow) using a suture relay technique to grasp the detached ligament together with the capsule. (F) Schema of the deltoid ligament repair. T: talus

The details of the surgical procedure are as follows: The patient was placed in the supine position under regional anesthesia, with sciatic nerve and saphenous nerve blocks administered, and without the use of a tourniquet. Traction was applied to increase the space in the ankle joint. Arthroscopy was performed through anteromedial, anterolateral, and accessory anteromedial portals. After gently detaching the deltoid ligament from the ossicular surface, the ossicle was resected, and the distal aspect of the medial malleolus was debrided (Figures 1A-1C).

One or two suture anchors were inserted into the distal aspect of the medial malleolus through the accessory anteromedial portal, positioned just distal to the medial malleolus. The suture wire was passed through the anterior edge of the detached ligament, subcutaneous tissue along the outer surface of the capsule, and the posterior edge of the detached ligament via a suture relay technique to grasp the detached ligament together with the capsule. After the termination of traction, the ligament and capsule were reattached to the distal aspect of the medial malleolus, while the ankle joint was kept in a neutral position (Figures 1D-1F).

Postoperative short-leg casting was applied for three weeks, with full weight-bearing permitted. After cast removal, walking with an arch-support insole and formal physical therapy were initiated. Running was allowed six weeks after surgery. Returns to play were allowed based on functional recovery. The following study data were retrospectively collected from medical charts: age, sex, follow-up period, symptom duration, and sports (Table 1).

**Table 1:** Characteristics of patients (three feet in three patients) AOFAS: American Orthopedic Foot and Ankle Society

Case	1	2	3
Age	15	16	12
Sex	M	F	M
Symptom duration (months)	48	3	4
Sport	soccer	gymnastics	soccer
AOFAS ankle-Hindfoot scale (preoperative)	77	54	57
AOFAS ankle-Hindfoot scale (last follow-up)	100	100	100
Time to start running (week)	6	9	8
Time to return to sports (week)	14	19	20
Follow-up (years)	3.5	8.8	7.9

The American Orthopedic Foot and Ankle Society (AOFAS) ankle-hindfoot scale score [9] was evaluated preoperatively and at the final follow-up. We also recorded the time to start running and the time to return to sports at the pre-injury level after surgery.

Case 1

**Figure 2:**
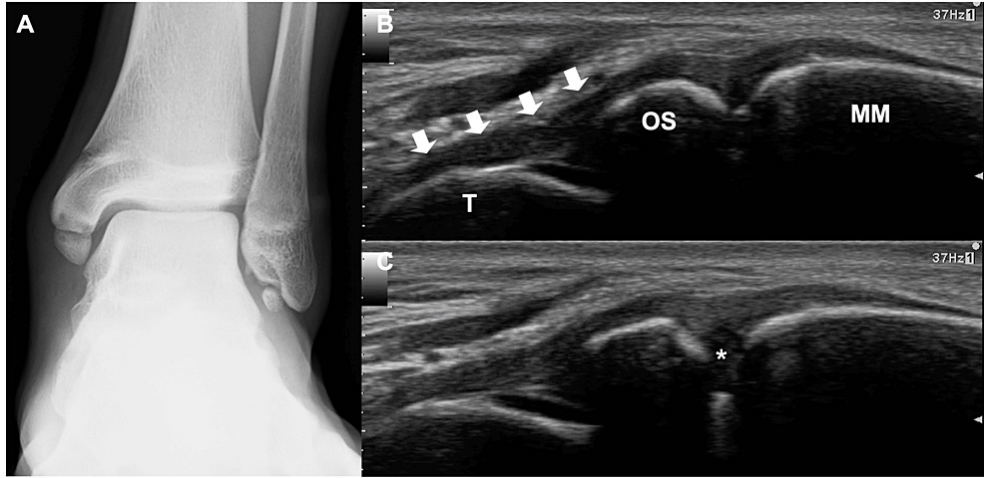
Preoperative radiograph and ultrasonographic images (A) Plain radiograph reveals a well-defined round-shaped bone beneath the medial malleolus (MM) and os subfibulare. (B) Ultrasonographic imaging reveals a superficial component of the deltoid ligament and the deep anterior tibiotalar ligament (arrows) attached to the ossicle (OS). (C) The gap (*) between the OS and the MM became wider while valgus stress was applied to the ankle. T: talar head

A 15-year-old male soccer player presented with a four-year history of pain in the left medial ankle. The ossicle of the medial malleolus was identified on plain radiography. As his pain disappeared one month after the cessation of sports activities, he returned to play. Since then, he experienced slight pain, although he could continue playing. However, the pain worsened, without any history of injury. The patient had no relevant medical history. Physical examination revealed tenderness in the distal medial malleolus. The range of motion of the ankle was normal. Plain radiography revealed a well-defined, round bony lesion beneath the medial malleolus (Figure 2A).

**Figure 3:**
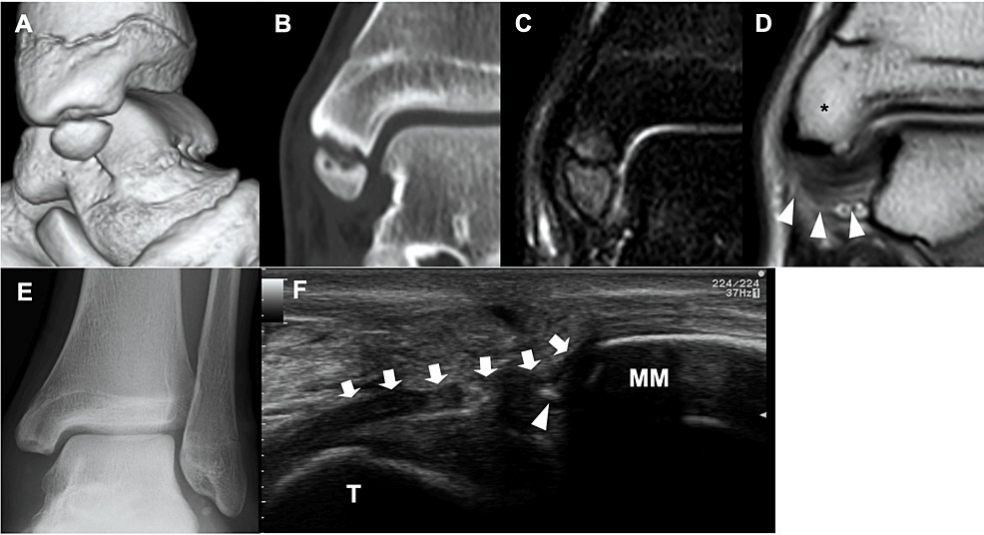
Pre- and postoperative images (A) Three-dimensional computed tomography (CT) shows almost the whole anterior colliculus and intercollicular groove of the medial malleolus is defective and the ossicle corresponds to this defective part. (B) Coronal plane CT reveals that there are subchondral sclerosis, cysts, and irregular cortex on each contact surface between the medial malleolus and ossicle. (C) The T2-weighted fat-suppressed image demonstrates that bone marrow edema is present on the ossicle and corresponding part of the medial malleolus. (D) Most part of the deep posterior tibiotalar ligament (arrowheads) is attached to the posterior colliculus of the medial malleolus (*). (E) Plain radiographs show that the ossicle was resected completely. (F) Postoperative US imaging shows that the superficial component of the deltoid ligament and the deep anterior tibiotalar ligament (arrows) were reattached to the medial malleolus by suture wire (arrowhead). T: talar head

Ultrasonographic imaging revealed the superficial component of the deltoid ligament and deep anterior tibiotalar ligament attached to the ossicle. When valgus stress was applied to the ankle, the ossicle showed instability (Figures 2B-2C). Computed tomography (CT) showed the detailed morphology of the medial malleolus and ossicle. Three-dimensional images revealed that almost the entire anterior colliculus and intercollicular groove of the medial malleolus were defective, and the ossicle (14.8 mm in length, 9.9 mm in width) corresponded to this defect. Multiplanar reconstruction images revealed subchondral sclerosis, cysts, and an irregular cortex on each contact surface between the medial malleolus and ossicle (Figures 3A-3B).

On MRI, T2-weighted fat-suppressed images revealed bone marrow edema in the ossicle and corresponding part of the medial malleolus. Proton density-weighted imaging revealed the anterior part of the deep posterior tibiotalar ligament, which is a component of the deltoid ligament, was attached to the posterior tip of the ossicle. However, most of the deep posterior tibiotalar ligament was found to be attached to the posterior colliculus of the medial malleolus (Figures 3C-3D). Since conservative treatment, including restriction of sports activity and physical therapy, was ineffective and the pain persisted, we performed surgical treatment. The preoperative AOFAS ankle-hindfoot scale score was 77. Postoperative plain radiography revealed a complete resection of the ossicle (Figure 3E). Postoperative ultrasonographic imaging revealed that the superficial and deep anterior tibiotalar ligaments of the deltoid ligament were reattached to the medial malleolus using the suture wire (Figure 3F). He started running six weeks after surgery and returned to sports 14 weeks postoperatively. No surgical complications occurred. The AOFAS ankle-hindfoot scale score at the final follow-up (3.5 years) was 100.

Case 2

**Figure 4:**
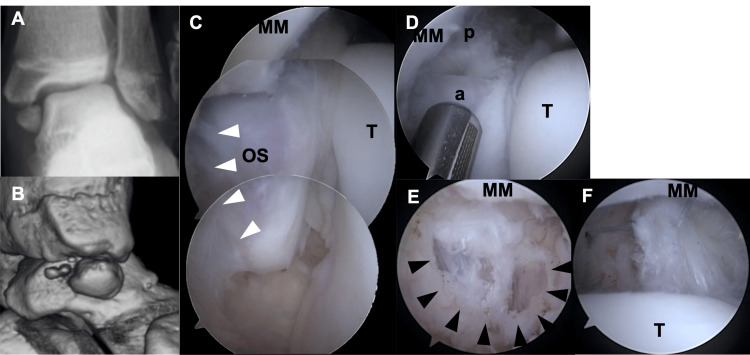
Preoperative images and arthroscopic findings (A) Valgus stress radiograph demonstrates widening of the gap between the medial malleolus (MM) and the ossicle (OS) and tilting of the talus. (B) Three-dimensional CT shows almost the whole anterior and posterior colliculus of the MM is defective and the OS corresponds to this defective part. The OS is composed of two separate bony parts. (C) The superficial component of the deltoid ligament (white arrowhead) is attached to the OS. The OS is clearly separated from the MM. (D) The OS is composed of an anterior large part (a) and a posterior small part (p). (E) The proximal end of the superficial component of the deltoid ligament is detached from the anterior large OS. (F) The proximal end of the deep posterior tibiotalar ligament detached from the posterior small OS is captured by repair suture wire. T: talus

A 16-year-old female gymnast presented with pain in the left medial ankle. The patient experienced medial ankle pain after landing while practicing gymnastics. Physical examination revealed tenderness in the distal medial malleolus. The patient was unable to squat due to medial ankle pain and had flatfoot. Plain radiography revealed a large, round bone beneath the medial malleolus. Valgus stress radiographs revealed a widening of the gap between the medial malleolus and ossicle, along with tilting of the talus, suggesting ankle instability (Figure 4A).

Three-dimensional CT revealed that almost all the anterior and posterior colliculi of the medial malleolus were defective, and the ossicle (13.8 mm in length, 9.7 mm in width) corresponded to this defect. The ossicle was composed of two separate bony parts (Figure 4B). Despite the three-month cessation of sports activities and physical therapy, the pain persisted, preventing her from returning to sports. Therefore, surgical treatment was performed. The preoperative AOFAS ankle-hindfoot scale score was 54. Arthroscopy revealed that most of the superficial components of the deltoid ligament and a portion of the deep posterior tibiotalar ligament were attached to the ossicle. This finding suggested that the ligaments needed to be detached for ossicle resection. In this case, two anchors were required to reattach the superficial component of the deltoid ligament and deep posterior tibiotalar ligament (Figures 4C-4F). She started running eight weeks after surgery and returned to sports 19 weeks postoperatively. The postoperative AOFAS ankle-hindfoot scale score at the final follow-up (8.8 years) was 100.

Case 3

**Figure 5:**
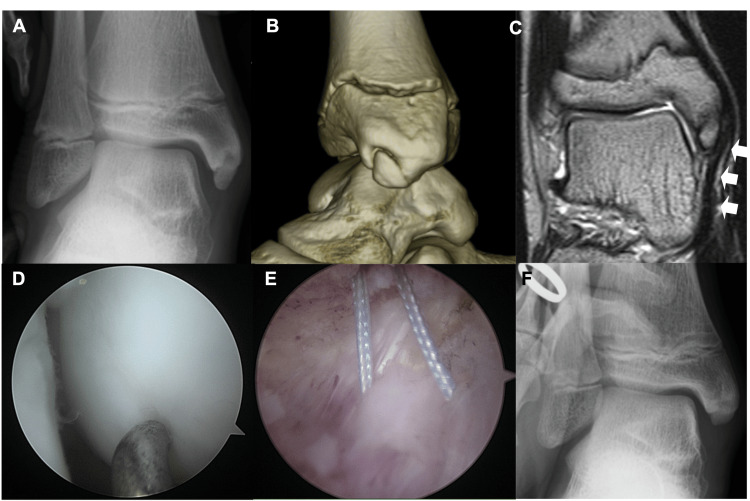
Pre- and postoperative images and arthroscopic findings of Case 3 (A) Valgus stress radiographs demonstrate that there are no instabilities (B) Three-dimensional CT shows anterior aspect of the anterior colliculus is defective and the ossicle (OS) corresponds to this defective part. (C) Magnetic resonance imaging (MRI) demonstrates that a superficial component of the deltoid ligament (DL) (white arrowhead) is attached to the OS. (D) Arthroscopy reveals that cartilaginous continuity between the medial malleolus (MM) and the OS is maintained. (E) The detached area of the DL is narrower than that of the other two cases. (F) Postoperative valgus stress radiographs demonstrate that stability of the ankle joint is maintained.

A 12-year-old male soccer player presented with pain in the right medial ankle. The patient experienced medial ankle pain after twisting his ankle while playing soccer. Three months of rest, including three weeks of short-leg-cast immobilization and physical therapy, were performed. However, the pain persisted, and the patient was referred to our hospital. Physical examination revealed tenderness in the distal medial malleolus. He also exhibited flatfoot. Valgus stress radiographs revealed no instability (Figure 5A).

Three-dimensional CT revealed that the anterior aspect of the anterior colliculus was defective, and the ossicle (7.7 mm in length, 5.9 mm in width) corresponded to this defect (Figure 5B). MRI revealed that the superficial component of the deltoid ligament was attached to the ossicle (Figure 5C). Additionally, bone marrow edema was identified in the ossicle and corresponding part of the medial malleolus. Surgery similar to that in the other two cases was performed. The preoperative AOFAS ankle-hindfoot scale score was 57. Arthroscopy revealed that the cartilaginous continuity between the medial malleolus and the ossicle was maintained, with softening of the cartilage between them facilitating the determination of the border (Figure 5D). The detached area of the deltoid ligament was narrower than that in the other two cases (Figure 5E). The patient started running nine weeks after surgery and returned to sports 20 weeks postoperatively. Postoperative valgus stress radiographs revealed that ankle joint stability was maintained (Figure 5F). The postoperative AOFAS ankle-hindfoot scale score at the final follow-up (7.9 years) was 100.

## Discussion

In the present three cases of symptomatic ossicles of the medial malleolus, we performed arthroscopic resections of the ossicles combined with deltoid ligament repairs. The main finding of the present case report was that the ligament attached to the ossicle varied in volume and type, depending on the case. Therefore, deltoid ligament repair is necessary based on each case. To the best of our knowledge, this is the first report to demonstrate the arthroscopic resection of the ossicle combined with deltoid ligament repair of symptomatic ossicles of the medial malleolus.

Several mechanisms have been proposed to explain the cause of pain, including synovitis and hypertrophic soft tissue impingement [6], impingement of the ossicles [7], traction apophysitis [1], osteochondrosis [10], and fibrous adhesion between the ossicle and medial malleolus, leading to false motion [2]. Among these, it is essential to differentiate traction apophysitis and osteochondrosis, as they can be treated conservatively, thereby avoiding unnecessary surgeries. From this perspective, prefused accessory ossification centers should be differentiated from other differential diagnoses such as avulsion fractures resulting in pseudoarthrosis, and accessory bones when considering surgical treatment. In Cases 1 and 2, stress-applied radiography or ultrasonography revealed instability of the ossicle, indicating that the ossicles were not accessory ossification centers of the medial malleolus. In contrast, in Case 3, the stress radiograph showed no instability, and arthroscopy revealed an unclear border between the ossicle and medial malleolus. These findings indicate the possibility that the ossicle may indeed be an accessory ossification center. However, it has been suggested that an accessory ossification center typically appears during the eighth and ninth years and fuses with the medial malleolus by the 11th year [11]. Considering the patient’s age and the ineffectiveness of conservative treatment for three months, surgical treatment was chosen. Although the origin of the ossicle may differ in each case, good short-term clinical outcomes were achieved in all cases.

Several surgical treatments have been reported for symptomatic ossicles of the medial malleolus. Kim et al. [2] reported that one of the two patients who underwent internal fixation resulted in nonunion, while the other patient who underwent open resection of the ossicle combined with deltoid ligament repair achieved good clinical outcomes. Teramoto et al. performed open reduction and internal fixation and reported good clinical outcomes in five patients. However, these surgeries were accompanied by bone grafting and included surgery in a nonunion case [4]. In addition, Taki et al. [3] reported that 6 of 10 patients who underwent internal fixation experienced nonunion. Based on these findings from previous reports, careful consideration of the indications for internal fixation of the symptomatic ossicle of the medial malleolus is necessary. In contrast, simple arthroscopic resection of the ossicle yielded good clinical results [6-8]. Shinohara et al. reported that they did not repair the deltoid ligament because the ossicle was easily removed from the deltoid ligament, and damage to the deltoid ligament was minimal [8]. In other reports, there was no detailed information on the ligament attached to the ossicle.

As a treatment for symptomatic ossicles of the lateral malleolus, known as os subfibulare, repair of the lateral ligament combined with resection of the ossicle has been performed to restore stability, given that the lateral ligament attaches to the ossicle [12-14]. Due to the lack of consensus on the surgical treatment for symptomatic ossicles of the medial malleolus, consideration of the ligament attached to the ossicle is crucial. Arthroscopy in the present cases revealed that the volume and type of ligament attached to the ossicle were different in each case, indicating that the presence of the ossicle affects the stability of the ankle joint and that the alignment of the foot differs in each case. The deltoid ligament is composed of two layers (superficial and deep), which are formed by multiple components. The attachment sites of each deltoid ligament component have been described in detail [15,16]. Based on these findings, the superficial component of the deltoid ligament and deep anterior tibiotalar ligament were considered to be on the ossicle in Cases 1 and 3. Furthermore, in Case 2, the attachment sites of all the components were considered to be involved. Amaha et al. described the attachment site of the capsular fibrous tissue to the medial malleolus in detail. Their findings also indicated that the capsular fibrous tissue of the medial ankle may be involved in the ossicle of the medial malleolus [17]. In other words, simple resection of the ossicle can cause deltoid ligament dysfunction. For instance, the superficial component of the deltoid ligament, which originates from the anterior colliculus of the medial malleolus, plays an important role in the pathophysiology of flatfoot [18,19]. In the present study, the patients in Cases 2 and 3 had flatfoot. Previous reports have described hyperpronated feet in patients [1,20]. In addition, simple resection of the ossicle poses an increased risk of ankle instability in cases with a large ossicle involving a wide attachment site to the deltoid ligament, as in Case 2. Furthermore, open resection of the ossicle can damage normal attachment sites of the medial malleolus. Repetitive sport-related mechanical stress is believed to cause this disease in athletes, as in the present case [1]. Therefore, simple resection of the ossicle in athletes can lead to an increased load on the remaining attachment site of the deltoid ligament, causing deltoid ligament failure. Thus, we firmly believe that the ossicle should be resected arthroscopically and the deltoid ligament should be repaired, especially in cases with large ossicles involving wide attachment sites of the deltoid ligament.

## Conclusions

In conclusion, we report three cases of symptomatic ossicles of the medial malleolus treated with arthroscopic resection of the ossicles combined with deltoid ligament repair. Surgery effectively improved patients’ symptoms and enabled them to return to sports early. Arthroscopic findings from the present three cases suggested that deltoid ligament repair is necessary based on each case, as the ligament attached to the ossicle varied in volume and type. Although there was no recurrence of the pain, the patients should continue with their follow-up sessions as there is a possibility of chronic medial ankle instability, flat foot deformity, or other complications.
